# Prevalence of Micronutrient Deficiency among People Living with HIV in Selected Rural Districts of the Eastern Cape Province of South Africa

**DOI:** 10.3390/nu15133017

**Published:** 2023-07-02

**Authors:** Ntombizodumo Nxasana, Kelechi Elizabeth Oladimeji, Guillermo-Alfredo Pulido-Estrada, Teke Ruffin Apalata

**Affiliations:** 1 Department of Laboratory Medicine and Pathology, Faculty of Health Sciences, Walter Sisulu University, Mthatha 5100, Eastern Cape, South Africa; zodumonxasana@gmail.com; 2 College of Graduate Studies, University of South Africa, Pretoria 0001, Gauteng, South Africa; 3 Department of Public Health, Faculty of Health Sciences, Walter Sisulu University, Mthatha 5100, Eastern Cape, South Africa; pulido762@yahoo.com

**Keywords:** prevalence, micronutrient deficiency, HIV, PLHIV, viral load suppression, geospatial, Eastern Cape, South Africa

## Abstract

Human immunodeficiency virus (HIV)/acquired immune deficiency syndrome (AIDS) leads to immune suppression, and micronutrients play vital roles in human immune responses. Hence, this study aimed to evaluate the effects of viral load suppression in adult HIV-infected patients receiving antiretroviral therapy (ART) on micronutrient deficiency and its prevalence in selected rural districts in the Eastern Cape Province of South Africa. This cross-sectional study was conducted from February 2019 to February 2021 among 50 consenting HIV-infected patients attending community health centers within the three selected districts. The data were analysed with ArcGIS v.10.8 to create geospatial maps; the Global Positioning System (GPS) for analysis and presentation; and SPSS version 25 for inferential statistics involving the *t*-test and Fisher’s exact test, with the level of significance set at *p* < 0.05. Of the 50 participants, a significant difference of *p* = 0.003 was observed in mean age among viral load-suppressed (42.9 ± 8.89 years) and unsuppressed (32 ± 6.3 years). In addition, significant differences in the mean viral load and CD4 counts (*p* < 0.05) were seen. Only iron micronutrient showed a statistically significant difference (*p* < 0.001) between the viral load-suppressed group (mean 14.8, SD 6.1) and the unsuppressed group (mean 8.1, SD 1.6). Of the 38 individuals from the OR Tambo district, overall micronutrient deficiency was 60.5% (13 (34.2%) deficient for zinc, 9 (23.7%) deficient for iron, 5 (13.2%) for folate, and 1 (2.63%) for vitamin D). In all three study districts, deficiencies in zinc, iron, and folate micronutrients exceeded 25%, particularly in those with an unsuppressed viral load. To address these micronutrient deficiencies, people living with HIV (PLHIV) require robust nutritional supplementation programs.

## 1. Introduction

Human immunodeficiency virus (HIV)/acquired immune deficiency syndrome (AIDS) remains a huge public health problem due to increased mortality and morbidity rates [1]. The past decades have witnessed an outstanding breakthrough in the availability of highly effective antiretroviral therapy, yet in 2021, 38.4 million people were estimated as living with HIV globally, two-thirds of whom are from the African Region, with 1.5 million newly infected cases and 650,000 HIV-related deaths [2,3]. The Eastern and Southern African regions carry the highest number of people living with HIV (PLHIV), new HIV infections, and AIDS-related deaths [4]. In South Africa, there were 7.5 million people living with HIV in 2020 and more than 83,000 AIDS-related deaths [4]. The HIV/AIDS pandemic is generally prevalent in vastly malnourished populations, and this is tangible in the case of sub-Saharan countries which carry the majority of PLHIV [5]. Despite the commendable stride in the growing number of people on antiretroviral therapy (ART), there is limited information about the links between viral load suppression and malnutrition [6].

Globally, over 800 million people are malnourished, while 1.5 to 2 billion have one or more chronic micronutrient deficiencies, particularly in calcium, iron, iodine, selenium, zinc, vitamin A, and folate [7,8,9]. Micronutrient deficiencies result from insufficient intake and absorption of vitamins and minerals to maintain good health and growth [10,11]. Malnutrition and poor micronutrient levels especially in PLHIV are associated with an increased risk of progression to AIDS [12,13,14]. HIV/AIDS affects nutritional status by increasing energy requirements, reducing food intake, and adversely affecting nutrient absorption and metabolism [15,16,17]. Malnutrition and food insecurity are associated with increased mortality and poor clinical outcomes among people living with HIV/AIDS [18,19]. HIV infection and malnutrition are part of a vicious cycle that contributes to immunodeficiency and negative health outcomes [20]. Malnutrition increases the risk of HIV pathogenesis, while HIV in turn activates malnutrition by reducing nutrient intake, absorption, and metabolism, which negatively affects the immune system. This process between malnutrition, the immune system, and HIV infection prompts dysfunctions within the immune system, makes the host to be more vulnerable to infection, and increases the severity of malnutrition [21,22]. Malnutrition may lead to reduced immunity and increased susceptibility to opportunistic infections, which can lead to further malnutrition. [15,23,24]. For guaranteeing that micronutrient needs are met, the World Health Organization (WHO) advocates increasing access to a diversified diet, food fortification, and micronutrient supplementation, predominantly in areas where micronutrient deficiencies are endemic [25]. The WHO and the United Nations Food and Agriculture Organization (FAO) have adopted four main strategies for improving dietary intake: food fortification, micronutrient supplementation, nutrition education, and disease control measures. The fortification of staple foods is key in improving dietary diversity and effectively decreasing micronutrient deficiencies [26].

With energy intakes ranging from very low in informal settlements to very high in urban centers, it is no surprise that micronutrient deficiencies are still highly prevalent, with the prevalence of malnutrition ranging from 13% to 78% globally and 45.4% in South Africa [27]. Despite national food fortification, the intake of numerous micronutrients remains low, particularly calcium, folate, and vitamins B, C, and D [28]. There is a dearth of literature on serum concentration levels of micronutrients in adult HIV-infected South Africans and their geospatial distribution, especially in the Eastern Cape Province (ECP). It is unfortunate that the extent of this challenge in our setting remains largely unknown, affecting the appropriate management of several thousands of HIV-infected patients on ART. This study aimed to determine the prevalence and geospatial distribution of micronutrient deficiency among adult PLHIV in selected rural districts of the Eastern Cape Province of South Africa.

## 2. Materials and Methods

### 2.1. Study Design, Setting, and Eligibility Criteria

This cross-sectional study was carried out among HIV-infected patients at seven health facilities in the three districts (OR Tambo; Joe Gqabi, and Alfred Nzo) of the Eastern Cape Province of South Africa over two years, from February 2019 to February 2021. Participants were enrolled based on the following inclusion criteria: (1) being a confirmed HIV-positive patient; (2) on ART for at least six months; (3) aged 18 years and above; and (4) being registered in one of the study sites. 

### 2.2. Sampling and Study Population

Multi-stage random sampling was used for selecting the study sites and population; in the first stage, three municipal districts (OR Tambo, Alfred Nzo, and Joe Gqabi) were selected from a total of six municipalities by a random draw. In stages two and three, four subdistricts and seven facilities were randomly selected, respectively. The study population comprised all HIV-infected patients 18 years of age and above on ART for at least six months who consented to participate in the study. A sub-cohort of 50 was drawn from a larger study population of 125. 

### 2.3. Data Collection

A validated WHO core and expanded stepwise questionnaire [29] was used for collecting data. The questionnaire was adapted for the context of this study. Sociodemographic data, medical history, anthropometric measurements, and bio-physiological measures, such as viral load and CD4 count, were accessed on patients’ records. Blood for determination of micronutrient deficiency was collected by venipuncture for laboratory analysis.

#### Blood Collection for Micronutrient Analysis

Whole blood was collected by the assistance of trained phlebotomists from participants into gel separator tubes for serum and into EDTA tubes for plasma. After collection, all samples were immediately transported in a cooler bag with ice packs to the laboratory, where whole blood for serum was allowed to clot and sera obtained by centrifugation, then separated into cryovial tubes and refrigerated at −80 °C until analysis. The whole blood for plasma was centrifuged, plasma-separated, and refrigerated until analysis. Plasma samples were collected for zinc analysis and serum samples for folate, iron, and vitamin D analysis.

### 2.4. Data Management and Analysis

Data were entered on Excel Microsoft 365 and cleaned using Statistical Package for Social Sciences (SPSS) version 22 and STATA version 15. For data analysis, both STATA v15 and SPSS v22 were used. Continuous variables were presented using mean with the standard deviation, while categorical variables were summarised using frequencies, percentage, and statistical graphs. The T-test and Fisher’s exact test were used to describe relationships between outcomes variables including viral load status and explanatory variables such as patients’ sociodemographic, clinical, and micronutrient profiles. The level of significance was set at <5% (*p*-value < 0.05) for statistical significance. 

#### 2.4.1. Micronutrient Analysis and Quantification

For folate, the Access Folate assay, a chemiluminescent immunoassay for the quantitative determination of folic acid levels in human serum was utilised using the Access Immunoassay Systems (Beckman Coulter, 2017, CA, United States). The Alinity c Iron assay was used for the direct colorimetric determination of iron in sera on the Alinity c analyser (Abbott Laboratories, IL, USA, 2017). For zinc determination, Inductively Coupled Plasma Mass Spectrometry (ICP-MS) was used. All ICP-MS experiments were performed on an Agilent 7900 ICP-MS(Agilent Research Laboratories, Santa Clara, CA, USA). The Access 25(OH) Vitamin D total assay, a paramagnetic particle, chemiluminescent immunoassay, was used for the quantitative determination of total 25-hydroxyvitamin D [25(OH) vitamin D] levels in sera using the Access 2 Immunoassay Systems.

#### 2.4.2. Geospatial Analysis of Micronutrient Deficiencies and Viral Load

**Figure 1:**
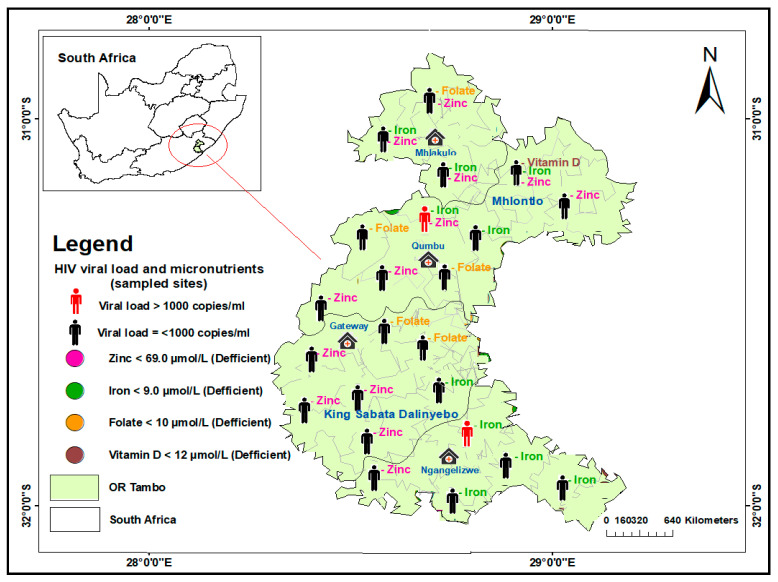
Distribution of HIV viral load suppression and non-suppression and selected micronutrient deficiencies in sampled sites of King Sabata Dalinyebo and Mhlontlo sub-districts in OR Tambo District, South Africa.

**Figure 2:**
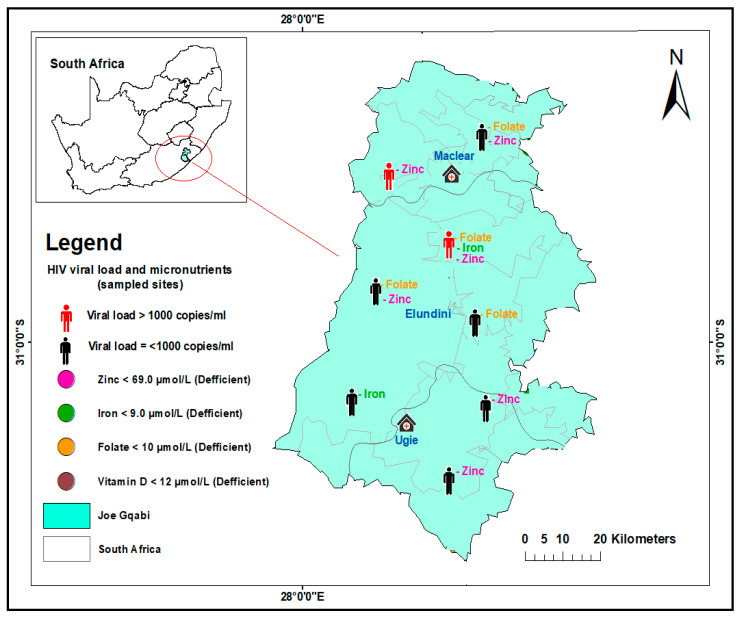
Distribution of HIV viral load suppression and non-suppression and selected micronutrient deficiencies in sampled sites of Elundini sub-district in Joe Gqabi District, South Africa.

**Figure 3:**
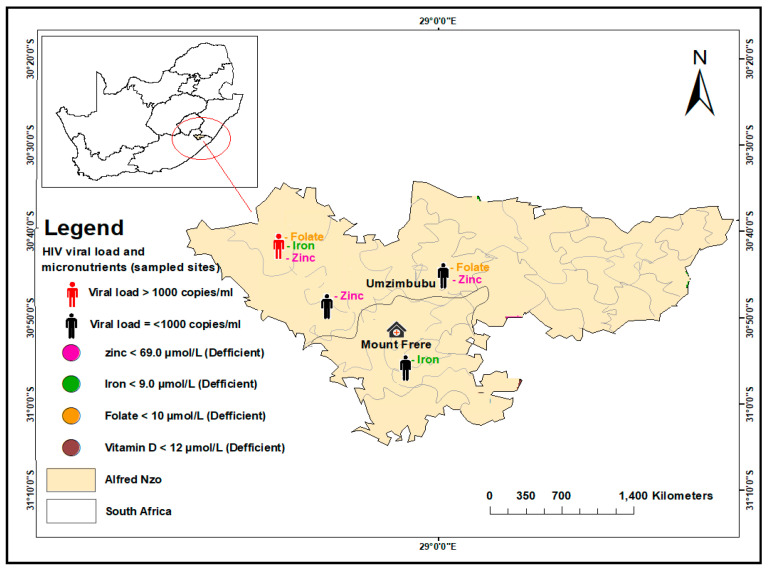
Distribution of HIV viral load suppression and non-suppression and selected micronutrient deficiencies in sampled sites of Umzimvubu sub-district in Alfred Nzo District, South Africa.

Geospatial maps were produced using ArcGIS v.10.8. The analysis was performed and presented using the Global Positioning System (GPS) to determine the sampled sites for the study. The distribution of HIV viral load non-suppression and micronutrient deficiencies for folate, zinc, iron, and vitamin D were populated on the spatial maps based on their coordinates in the sub-districts and facilities where the sampling was carried out. The areas covered were King Sabata Dalindyebo (KSD) and Mhlontlo sub-districts of OR Tambo district, Umzimvubu in Alfred Nzo district, and Elundini in Joe Gqabi district, as presented in Figure 1, Figure 2 and Figure 3.

## 3. Results

### 3.1. Sociodemographic and Clinical Characteristics

Of the 50 participants, a significant difference of *p* = 0.003 was observed in mean age among viral load-suppressed (42.9 ± 8.89 years) and unsuppressed (32 ± 6.3 years). Regarding other sociodemographic variables included in this study, none showed statistically significant relationships with the viral load, district (*p* = 0.240), sub-district of residence (*p* = 0.348), employment status (*p* = 0.684), income (*p* > 0.999), and gender (*p* > 0.999) (Table 1). Clinically, the mean CD4 count showed a significant difference (*p* = 0.000) between the two groups as well as in the CD4 categories of <200 to >350 (Table 2). The association of BMI, dyslipidaemias, and a previous history of some diseases (hypertension, diabetes, and hepatitis B infection) with the viral load was not significant (Table 2). 

### 3.2. Micronutrient Deficiency Patterns

A statistically significant difference (*p* < 0.001) was observed in means of iron micronutrient between the viral load-suppressed and unsuppressed groups. The other micronutrients were not statistically associated with viral load suppression, vitamin D (*p* = 0.629), folate (*p* = 0.614), and zinc (*p* = 0.805) (Table 3). We further analysed micronutrient patterns according to the three sampled districts; zinc showed the highest deficiency across the three districts, followed by iron (Table 4).

### 3.3. Geospatial Distribution of Micronutrient Deficiency

In the OR Tambo district, out of 38 participants analysed for micronutrient deficiency, 23 (60.5%) were micronutrient-deficient, 13 (34.2%) deficient for zinc, 9 (23.7%), deficient for iron, 5 (13.2%) for folate, and 1 (2.63%) for vitamin D. Twelve participants resided in the sub-district of King Sabata Dalindyebo (KSD), with five deficient in zinc, five in iron, and two in folate. In the Mhlontlo sub-district, there were eleven (28.9%) deficiencies with zinc being the most deficient in eight participants. Of these eight, five showed combinations of two micronutrients including one VL non-suppressed participant, and one showed three combinations, with zinc featuring in all combinations. (Figure 1). A total of 50% of iron-deficient participants resided in the southeastern part of the OR Tambo district, in the KSD subdistrict, which is more peri-urban. Another 50% resided in the northern part of the district, which is rural, coupled with all participants with two or three combinations of micronutrient deficiencies. A total of 84.6% of zinc-deficient participants were found from the mid-western to the western part of the OR Tambo district, from south to north.

At the Elundini sub-district of Joe Gqabi district, micronutrient deficiency was evident in eight (100%) of the participants. Of the eight participants, two were VL-unsuppressed, and of the two, one was deficient in three micronutrients. The two combinations of deficient micronutrients were seen in VL-suppressed (Figure 2).

In the Umzimvubu sub-district of Alfred Nzo district, four (100%) participants showed deficiencies in micronutrients. Of the four, one was VL non-suppressed and deficient in three micronutrients. Of the other three participants, one had a combination of two deficient micronutrients, and two had a single deficiency (Figure 3).

## 4. Discussion

Although ART may not compensate for micronutrient deficiencies in PLHIV, it does boost the immune system, so monitoring nutritional deficiencies may aid in tracking how well an HIV patient responds to treatment. The study findings revealed a prevalence of micronutrient deficiencies ranging from 60% to 100% across the three districts among PLHIV who were on ART for at least six months. Combinations of two or three micronutrient deficiencies were not uncommon in the participants. A significant age difference (*p* = 0.003) was observed among viral load-suppressed (42.9 ± 8.89 years) and unsuppressed (32 ± 6.3 years) where viral non-suppression was observed among younger participants. A study carried out in Canada reported similar results of a lower proportion of young adults achieving viral suppression compared to older adults [30]. According to a recent South African national HIV survey, older adults have the highest rate of viral suppression (73.2%), while younger adults (15–24) have the lowest [29]. A systematic review and meta-analysis also found that young adults and adolescents had significantly lower rates of HIV viral suppression after starting ART [31]. Clinical outcomes tend to be suboptimal in subpopulations of adults younger than 29 years old living with HIV, as reported by Agwu and Ryscavage in 2011 [32,33]. Moreover, many challenges youth face can lead to treatment non-adherence and poor retention in care, putting them at risk of viral rebound [34]. 

Deficiencies above 25% were seen for zinc, iron, and folate micronutrients in all three districts, especially in those with unsuppressed viral load. Among the three sampled districts, zinc showed the highest deficiency across the districts, followed by iron. OR Tambo district had a micronutrient deficiency of 60.5%, and both Joe Gqabi and Alfred Nzo had a deficiency of 100%. Among the three geographic districts studied, the main differences in socioeconomic position are that Joe Gqabi has the most land area, while OR Tambo has the largest number of residents, accounting for 26.2% of the provincial population. The unemployment rate in the Alfred Nzo district is 39.73%, followed by OR Tambo at 37.7%. Alfred Nzo District is the poorest in the province, with a poverty rate of 71.5%, followed by OR Tambo at 66.5% and Joe Gqabi at 55.6%. Furthermore, HIV/AIDS remained the leading cause of death for people aged 15–24 and 25–64 in all three districts in 2019. HIV/AIDS prevalence in the 15–24 age group ranged between 18 and 22%, and in the 25–64 age group ranged between 24 and 28%. The OR Tambo district had the highest HIV/AIDS prevalence in the 25–64 age group, at 27.7% [34]. Much of the study sample was clustered in the OR Tambo region due to the large number of consenting participants. 

Micronutrient deficiencies have been linked to accelerated HIV disease progression and death. Thus, micronutrient adequacies are essential in maintaining a responsive immune system [35,36]. Throughout the course of HIV infection, multiple nutrient deficiencies manifest themselves relatively early. They are brought on by nutrient storage problems, malabsorption, altered metabolism, gut infections, altered gut barrier function, chronic diarrhea, anorexia, etc. [3] When compared to non-HIV patients, the prevalence of nutrient abnormalities is higher in HIV-infected patients. When compared to healthy people, HIV-positive patients appear to need multiples of the recommended dietary allowances for these nutrients [12,35,36,37,38]. We noted the lack of studies as a challenge in discussing the implications of our study findings as summarised above in the available published evidence. For instance, the only relevant national evidence in South Africa is the South African National Health and Nutrition Examination Survey (SANHANES-1) conducted in 2013. At the time of writing this study report, the SANHANES-2 event is anticipated to occur later in 2023 [37]. Furthermore, unlike the present study, the SANHANES-1 examined micronutrient deficiency through dietary intake frequency and recall determining dietary diversity rather than through laboratory analysis of blood samples. Additionally, the dietary diversity assessment was performed on children rather than adults living with HIV [23,24,25,26,27,28,29,35,36,37,38]. Despite the absence of data to compare with the current study, one statement made in the SANHANES-1 report that is consistent with our study’s findings is that the typical South African diet is high in energy but low in micronutrients [38]. It also discovered no differences in the diversity of micronutrients between urban and rural areas. Except for folate, there was no difference in micronutrient deficiencies across our study’s districts, some of which were more rural and peri urban. Evidence reports that folate deficiency slows down DNA synthesis and cell division, which has an impact on bone marrow and other areas with high rates of cell turnover [35,39]. 

This study is one of the few studies to show the geospatial distribution of micronutrient deficiency especially in the Eastern Cape Province of South Africa. However, the limitation of the study was the small sample size. We recommend for researchers to consider replicating the study with a larger sample size and assessment of advanced causal relationships between micronutrient deficiencies and immune response. 

## 5. Conclusions

The findings of this study suggest that more research is needed to overcome viral non-suppression, particularly among young adults. Furthermore, regardless of viral load status, micronutrient deficiencies were found in all three sampled districts. Poverty is an important contributor to malnutrition, as previously stated, and the sampled district has a low socioeconomic status. When combined with antiretroviral therapy, nutritional interventions may help improve nutritional and clinical outcomes. As a result, we advocate for increased government monitoring and evaluation of HIV supplementation programs. Furthermore, informing PLHIV of their micronutrient status is an important part of the immediate, targeted interventions that should be implemented to improve PLHIV nutritional status and reduce the impact of HIV/AIDS.

## Figures and Tables

**Table 1:** Sociodemographic characteristics of the study population.

Sociodemographic Characteristics	VL < 1000 (*n* = 43)	VL ≥ 1000 (*n* = 7)	Total (*n* = 50)	*p*-Value
Age ^a^	42.9 ± 8.9	32 ± 6.3	41.4 ± 9.3	0.003 ^c^
District ^b^				
Alfred Nzo	3 (7.0)	1 (14.3)	4 (8.0)	0.240 ^d^
Joe Gqabi	6 (14.0)	2 (28.6)	8 (16.0)	
Oliver R Tambo	34 (79.1)	4 (57.1)	38 (76.0)	
Sub-district ^b^				
KSD	17 (39.5)	3 (42.9)	20 (40.0)	0.348 ^d^
Elundini	6 (14.0)	2 (28.6)	8 (16.0)	
Mhlontlo	17 (39.5)	1 (14.3)	18 (36.0)	
Mzimvubu	3 (7.0)	1 (14.3)	4 (8.0)	
Employment ^b^				
Employed	22 (55.0)	4 (66.7)	26 (56.5)	0.684 ^d^
Unemployed	18 (45.0)	2 (33.3)	20 (43.5)	
Income ^b^				
<R5500	32 (80.0)	5 (83.3)	37 (80.4)	>0.999 ^d^
≥R5500	8 (20.0)	1 (16.7)	9 (19.6)	
Gender ^b^				
Female	42 (97.7)	7 (100.0)	49 (98.0)	>0.999 ^d^
Male	1 (2.3)	0 (0.0)	1 (2.0)	

^a^ Mean ± SD; ^b^ Frequency (%); ^c^ T test for independent samples; ^d^ Fisher’s exact test.

**Table 2:** Clinical characteristics of the study population.

Clinical Characteristics	VL < 1000 (*n* = 43)	VL ≥ 1000 (*n* = 7)	Total (*n* = 50)	*p*-Value
BMI ^a^	31.6 ± 7.5	26.7 ± 6.2	31.0 ± 7.5	0.135 ^c^
BMI categorised ^b^				
Underweight	0 (0.0)	1 (16.7)	1 (2.3)	0.081 ^d^
Normal weight	4 (10.8)	1 (16.7)	5 (11.6)	
Overweight	14 (37.8)	3 (50.0)	17 (39.5)	
Obesity	19 (51.4)	1 (16.7)	20 (46.5)	
Dyslipidaemia ^a^				
Triglycerides	1.39 ± 0.68	1.12 ± 0.23	1.35 ± 0.64	0.339 ^c^
HDL	1.39 ± 0.36	1.06 ± 0.21	1.34 ± 0.36	0.036 ^c^
LDL	2.18 ± 0.97	1.69 ± 0.47	2.11 ± 0.92	0.237 ^c^
CD4 ^a^	620.7 ± 236.4	301.7 ± 362.4	576 ± 276.5	0.004 ^c^
CD4 categorised ^b^				
<200	1 (2.3)	4 (57.1)	5 (10.0)	0.000 ^d^
200–350	4 (9.3)	1 (14.3)	5 (10.0)	
>350	38 (88.4)	2 (28.6)	40 (80.0)	
Medical history ^b^				
Diabetes				
Yes	2 (8.3)	0 (0.0)	2 (7.7)	>0.999 ^d^
No	22 (91.7)	2 (100.0)	24 (92.3)	
Hypertension				
Yes	11 (28.2)	0 (0.0)	11 (24.4)	0.311 ^d^
No	28 (71.8)	6 (100.0)	34 (75.6)	
Co-infection with hepatitis				
Positive	4 (10.0)	0 (0.0)	4 (8.9)	>0.999 ^d^
Negative	36 (90.0)	5 (100.0)	41 (91.1)	

^a^ Mean ± SD; ^b^ Frequency (%); ^c^ T test for independent samples; ^d^ Fisher’s exact test.

**Table 3:** Relationship between micronutrients and viral load status.

Micronutrients	VL < 1000 (*n* = 43)	VL ≥ 1000 (*n* = 7)	Total (*n* = 50)	*p*-Value
Vitamin D ^a^	25.8 ± 8.5	23.6 ± 11.1	25.5 ± 8.8	0.608 ^c^
Vitamin D categorised ^b^				
<12 ng/mL	1 (2.9)	0 (0.0)	1 (2.6)	0.629 ^d^
12–19 ng/mL	7 (20.6)	2 (40.0)	9 (23.1)	
≥20 ng/mL	26 (76.5)	3 (60.0)	29 (74.4)	
Folate ^a^	16.5 ± 10.2	14.5 ± 8.1	16.2 ± 9.9	0.653 ^c^
Folate categorised ^b^				
<10 nmol/L	9 (22.0)	2 (33.3)	11 (23.4)	0.614 ^d^
≥10 nmol/L	32 (78.0)	4 (66.7)	36 (76.6)	
Iron ^a^	14.8 ± 6.1	8.1 ± 1.6	14.0 ± 6.1	0.000 ^c^
Iron categorised ^b^				
<9	10 (25.0)	4 (66.7)	14 (30.4)	0.060 ^d^
9–30	30 (75.0)	2 (33.3)	32 (69.6)	
Zinc ^a^	76.9 ± 30.8	62.7 ± 16.4	75.0 ± 29.5	0.241 ^c^
Zinc categorised ^b^				
<69 µg/L	18 (41.9)	4 (57.1)	22 (44.0)	0.805 ^d^
69–149 µg/L	22 (51.2)	3 (42.9)	25 (50.0)	
>149 µg/L	3 (7.0)	0 (0.0)	3 (6.0)	

^a^ Mean ± SD; ^b^ Frequency (%); ^c^ T test for independent samples; ^d^ Fisher’s exact test.

**Table 4:** Micronutrients.

Micronutrients	OR Tambo	Joe Gqabi	Alfred Nzo	*p*-Value
Vitamin D ^a^	24.9 ± 8.5	28.8 ± 11.0	25.0 ± 8.6	0.614 ^c^
Vitamin D categorised ^b^				
<12 ng/mL	1 (3.4)	0 (0.0)	0 (0.0)	>0.999 ^d^
12–19 ng/mL	7 (24.1)	1 (16.7)	1 (25.0)	
≥20 ng/mL	21 (72.4)	5 (83.3)	3 (75.0)	
Folate ^a^	18.1 ± 10.3	10.3 ± 6.4	9.3 ± 2.7	0.053 ^c^
Folate categorised ^b^				
<10 nmol/L	5 (13.9)	4 (57.1)	2 (50.0)	0.015 ^d^
≥10 nmol/L	31 (86.1)	3 (42.9)	2 (50.0)	
Iron ^a^	13.2 ± 5.4	18.9 ± 8.9	13.8 ± 6.0	0.101 ^c^
Iron categorised ^b^				
<9	10 (27.8)	2 (33.3)	2 (50.0)	0.724 ^d^
9–30	26 (72.2)	4 (66.7)	2 (50.0)	
Zinc ^a^	79.7 ± 32.1	59.0 ± 11.1	61.8 ± 7.7	0.128 ^c^
Zinc categorised ^b^				
<69 µg/L	13 (34.2)	6 (75.0)	3 (75.0)	0.207 ^d^
69–149 µg/L	22 (57.9)	2 (25.0)	1 (25.0)	
>149 µg/L	3 (7.9)	0 (0.0)	0 (0.0)	

^a^ Mean ± SD; ^b^ Frequency (%); ^c^ T test for independent samples; ^d^ Fisher’s exact test.

## Data Availability

Data from this study will be made available from the corresponding author upon request.
